# Long-Term Efficacy of *Limosilactobacillus reuteri* DSM17938 in the Prevention of Functional Abdominal Pain Disorders

**DOI:** 10.3390/nu18040687

**Published:** 2026-02-20

**Authors:** Flavia Indrio, Antonio Di Mauro, Giacomo Perrone, Annamaria Greco, Simona Filoni, Enea Vincenzo Napolitano, Luca Pecoraro

**Affiliations:** 1 Department of Experimental Medicine, Pediatric Section, University of Salento Hospital “Vito Fazzi”, 73100 Lecce, Italy; 2 Pediatric Primary Care, National Pediatric Health Care System, ASL Bari, 70100 Bari, Italy; 3 Pediatric Unit, Ospedale Vito Fazzi, ASL Lecce, 73100 Lecce, Italy; 4 Department of Electrical Engineering and Information Technology, University of Naples Federico II, 80125 Naples, Italy

**Keywords:** probiotics, functional gastrointestinal disorders, irritable bowel syndrome, functional abdominal pain

## Abstract

Introduction: Pediatric Functional Gastrointestinal Disorders (FGIDs), including infantile colic and constipation, may persist into later childhood and adulthood, sometimes manifesting as functional abdominal pain (FAP). Early exposure to probiotics during critical developmental windows may influence long-term susceptibility to disease. Background/Objectives: Building on our original randomized controlled trial, which demonstrated that *Lactobacillus reuteri* DSM 17938 reduced acute infantile FGID symptoms, a 10-year follow-up study was performed to evaluate whether this early intervention provided lasting protection against FAP in childhood. Methods: Two hundred participants from the original RCT cohort completed follow-up assessments at age ten. The primary outcome was the presence of FAP, analyzed according to the original randomization group (probiotic vs placebo). FAP was diagnosed at age 10 using the Rome IV criteria, based on a standardized clinical assessment by a pediatric gastroenterologist who was blinded to the original allocation. Results: FAP was diagnosed in 13/99 (13.1%) children in the probiotic group and 81/101 (80.2%) in the placebo group, corresponding to an absolute risk reduction of 67.1% (95% CI 56.8–77.3) and a relative risk of 0.16 (95% CI 0.10–0.27) (*p* < 0.001). Conclusions: Early supplementation with *L. reuteri* DSM 17938 was associated with a markedly lower prevalence of FAP at age 10. However, the long-term follow-up was observational and characterized by a 57.2% attrition rate. In addition, longitudinal data on potential confounders were unavailable; therefore, the findings should be interpreted as an association rather than proof of causality.

## 1. Introduction

The management of chronic functional gastrointestinal disorders (FGIDs) represents a persistent challenge in children [1,2]. These disorders are defined by recurrent gastrointestinal symptoms without identifiable structural or biochemical abnormalities and typically originate early in life, with manifestations such as infantile colic, regurgitation, and constipation appearing within the first months after birth [1,2]. The developmental continuity observed across age groups suggests that FGIDs may originate from early alterations in gut physiology, immune maturation, or neurogastroenteric signaling [3]. Increasing evidence indicates that environmental exposures during the first 1000 days of life—a critical window characterized by marked biological plasticity—play a pivotal role in shaping long-term health outcomes [4]. Factors such as antibiotic exposure, mode of delivery, feeding practices, and maternal health can influence early microbial colonization patterns. Deviations from typical colonization trajectories may result in sustained changes in microbial diversity and metabolic function, potentially affecting immune regulation and increasing susceptibility to FGIDs later in life [4,5]. Nevertheless, the pathophysiology of FGIDs is widely recognized as multifactorial. Beyond microbial influences, psychosocial stressors, visceral hypersensitivity, genetic predisposition, and alterations in gut–brain communication contribute to disease onset and persistence. These mechanisms likely interact dynamically throughout development rather than acting in isolation [4,5]. Longitudinal studies have linked early-life microbial dysbiosis not only to subsequent FGIDs but also to allergic and neurobehavioral disorders, supporting the concept of early microbial programming [3]. Within this framework, interest has grown in interventions that modulate early microbial and host development. Probiotics have been investigated as a strategy to influence gut–brain–immune interactions during infancy. Available evidence suggests that selected strains may alleviate acute gastrointestinal symptoms and, potentially, exert longer-term effects extending into childhood [6]. Mechanistically, certain probiotics have been shown to modulate immune responses, strengthen epithelial barrier integrity, and influence neural pathways involved in pain perception and stress responsiveness [6]. Emerging data also suggest possible associations with neurodevelopmental outcomes [7] and reduced incidence of FGIDs in later years [8]. However, the current body of longitudinal evidence remains heterogeneous with respect to study design, population characteristics, microbial assessment techniques, and clinical endpoints. Many studies are limited by modest sample sizes, variable follow-up durations, and inconsistent findings, thereby constraining causal interpretation and highlighting the need for well-designed long-term investigations. Regarding *Limosilactobacillus reuteri* DSM 17938, experimental and clinical data suggest that this strain may modulate gut microbial composition and interact with intestinal mucosal signaling pathways, contributing to improved gastrointestinal motility and enhanced pain tolerance [9]. These mechanisms are considered relevant to the pathophysiology of infantile colic, regurgitation, functional abdominal pain, and functional constipation in pediatric populations [9]. Despite promising short-term findings, relatively few studies have examined whether early probiotic supplementation is associated with sustained reductions in FGIDs over extended follow-up. Most available trials focus on symptom relief during infancy, with limited data extending into later childhood. This gap underscores the need for long-term prospective observational studies to determine whether early microbial modulation can truly alter disease trajectories rather than merely delay symptom onset. In light of this unmet need, the present prospective observational study was conducted as a 10-year follow-up of a previous randomized, double-blind, placebo-controlled trial involving 468 healthy infants who received *Limosilactobacillus reuteri* DSM 17938 or placebo during the first three months of life [10]. While the original trial evaluated short-term gastrointestinal outcomes in infancy, the current investigation aimed to assess the long-term association between early probiotic exposure and the prevalence of FAP in later childhood.

## 2. Materials and Methods

**Figure 1:**
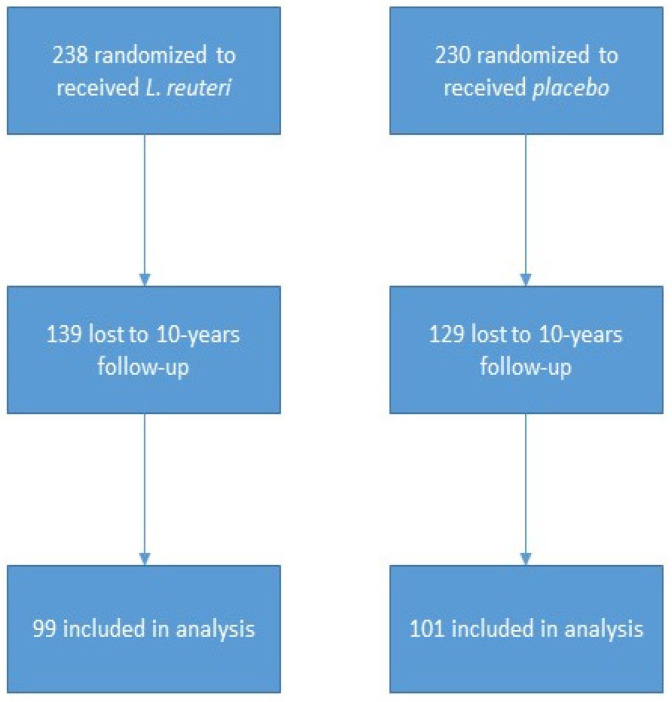
Children enrolled in the study and the total number assessed.

This prospective 10-year observational follow-up study was derived from an original randomized, double-blind, placebo-controlled trial, in which 468 infants were assigned during the first three months of life to receive either *L. reuteri* DSM 17938 (BioGaia AB, Stockholm, Sweden) (n = 238) or a placebo (n = 230) [10]. Of the 468 randomized participants, 200 (42.8%) completed the 10-year assessment, corresponding to an overall attrition rate of 57.2% (268/468). Loss to follow-up was primarily due to unsuccessful re-contact, family relocation, refusal to participate, or incomplete assessment. However, detailed reasons were not systematically documented. Given the prolonged follow-up and the substantial attrition rate, residual confounding and selection bias cannot be excluded. To explore the potential impact of attrition, baseline characteristics of participants who completed the 10-year assessment were compared according to original treatment allocation and sex. The participant flow is summarized in detail in Figure 1.

To determine whether participants who completed follow-up differed systematically from those lost to follow-up, we examined the association between follow-up participation and the original randomization group, as well as sex, using chi-square tests. At the 10-year assessment, clinical data were collected to establish the prevalence of functional abdominal pain (FAP). All participants underwent a standardized clinical evaluation performed by an experienced pediatric gastroenterologist who remained blinded to the original treatment allocation. Diagnoses were made according to the updated Rome IV criteria for pediatric functional gastrointestinal disorders, ensuring consistency with internationally recognized definitions [11]. According to Rome IV criteria, FAP is defined as abdominal pain occurring at least four times per month over a minimum period of two months, not exclusively related to physiological events such as eating or menstruation, and not fulfilling criteria for other functional gastrointestinal disorders, including irritable bowel syndrome, functional dyspepsia, or abdominal migraine. In the absence of alarm features, the diagnosis is based on symptoms and does not require extensive diagnostic testing [7]. During clinical evaluation, children were systematically assessed for red flags suggestive of organic disease (e.g., weight loss, persistent vomiting, gastrointestinal bleeding, growth failure, nocturnal symptoms, or a family history of inflammatory bowel disease or celiac disease). When clinically indicated, additional investigations were performed in accordance with standard practice. The primary analysis evaluated the association between the original intervention (probiotic vs. placebo) and the presence of FAP at 10 years of age. Associations between categorical variables were assessed using chi-square tests, with effect sizes quantified by Cramér’s V. Absolute and relative effect measures were calculated, including absolute risk reduction (ARR), relative risk (RR), and number needed to treat (NNT). To examine the robustness of the primary findings, an exploratory multivariable logistic regression model was constructed. FAP at 10 years (yes/no) was entered as the dependent variable, and original treatment allocation was the primary independent variable. The model was adjusted for baseline characteristics collected during infancy, including sex, feeding category, and markers of early symptom burden and healthcare utilization (colic episodes, regurgitation episodes, daily crying duration, and pediatrician visits). Because longitudinal data on time-varying confounders over the 10-year follow-up (e.g., antibiotic exposure, diet, psychosocial stress, or intercurrent infections) were unavailable, residual confounding cannot be excluded. Primary outcome analyses were conducted using a complete-case approach among participants with available 10-year data (modified intention-to-treat framework). The extent of missing outcome data is reported by treatment group, and no imputation procedures were applied. Statistical significance was defined as a two-sided alpha level of 0.05. A secondary exploratory analysis assessed the distribution of FAP according to early infant feeding practices. Participants were categorized as exclusively formula-fed or partially/fully breastfed. The latter categories were combined to ensure adequate subgroup size and statistical stability. Feeding information was prospectively collected during infancy as part of the original trial, thereby minimizing recall bias. Prevalence rates were calculated for each group, and associations were tested using chi-square analysis without adjustment for multiple comparisons, given the exploratory nature of this analysis. Statistical analyses were performed using Python (version 3.11.4, Python Software Foundation, Wilmington, DE, USA).

## 3. Results

Baseline characteristics were compared between intervention groups among participants who completed the 10-year follow-up, including the sex distribution and infant feeding type (Table 1).

The analysis of the 10-year follow-up cohort included an evaluation of potential selection and attrition bias related to loss to follow-up. Participation at 10 years (participants vs non-participants) was not significantly associated with original treatment allocation (*p* = 0.3034, Cramér’s V = 0.048) or sex (*p* = 0.6968, Cramér’s V = 0.018), indicating no detectable imbalance across these baseline variables. To further explore the possibility of selection bias, baseline infant characteristics were compared between participants who completed the 10-year assessment and those lost to follow-up (Table 2).

Follow-up participation was not associated with original treatment allocation or sex. However, responders exhibited a lower baseline symptom burden, characterized by fewer colic episodes and fewer daily crying minutes, as well as a lower proportion of missing infant feeding data. These findings indicate that selective participation based on early symptom severity cannot be ruled out and may affect the generalizability of absolute prevalence estimates. Within the responder cohort, the probiotic and placebo groups remained well balanced; no significant association was observed between sex and treatment allocation (*p* = 0.2059, Cramér’s V = 0.089). The two groups (probiotic, n = 99; placebo, n = 101) were subsequently compared with respect to the presence of FAP at 10 years of age. Outcome data for FAP were available for 193 of the 200 participants. Seven assessments were missing: five in the probiotic group and two in the placebo group. In complete-case analyses, FAP was diagnosed in 13 of 94 children (13.8%) in the probiotic group compared with 81 of 99 children (81.8%) in the placebo group (Table 3).

This corresponded to an absolute risk reduction of 68.0% (95% CI, 56.8–77.3), a relative risk of 0.16 (95% CI, 0.10–0.27), and a number needed to treat of 1.5. The association between treatment allocation and FAP was highly statistically significant (*p* < 0.001) and was characterized by a large effect size (Cramér’s V = 0.67). In absolute terms, FAP occurred in 13.1% (13/99) of children in the probiotic group compared with 80.2% (81/101) in the placebo group. Consistent with the absence of an association between sex and treatment allocation, sex was not significantly associated with FAP occurrence (*p* = 0.2214, Cramér’s V = 0.123), suggesting that sex was unlikely to confound the primary analysis meaningfully. Regarding the secondary analysis, the prevalence of FAP across infant feeding categories—exclusively formula-fed and partially/fully breastfed—ranged narrowly between 47% and 50%. No significant association was observed between feeding type and FAP at follow-up (chi-square test, *p* = 0.198).

## 4. Discussion

In an exploratory multivariable logistic regression model adjusted for baseline covariates (sex, infant feeding category, and markers of early symptom burden and healthcare utilization), early probiotic supplementation remained strongly associated with lower odds of FAP at 10 years (adjusted odds ratio ≈ 0.04; *p* < 0.001) (Table 4).

None of the baseline indicators of symptom severity showed an independent association with FAP in the adjusted model. This long-term prospective follow-up study demonstrates that early administration of *Lactobacillus reuteri* DSM 17938 was associated with a lower incidence of FAP at ten years of age (*p* < 0.001, Cramér’s V = 0.673). The magnitude of this association, reflected by a large effect size, should be interpreted cautiously. Such high values are uncommon in long-term observational studies and should be interpreted cautiously. Several factors may have contributed to this finding. First, the outcome was dichotomous and highly polarized between the probiotic and placebo groups, which can exaggerate Cramér’s V compared with effect size measures from continuous outcomes. Second, the intervention occurred during a sensitive developmental window, during which early-life exposures can exert lasting effects on gut–brain axis maturation, potentially amplifying differences in susceptibility to functional disorders. Residual confounding cannot be entirely excluded. Although the original trial was randomized and the responder cohort remained balanced for key baseline variables, unmeasured factors—such as antibiotic exposure, intercurrent infections, psychosocial stress, dietary patterns, and healthcare-seeking behavior over 10 years—may have affected the observed association. The high prevalence of FAP in the placebo group (80.2%) likely reflects the characteristics of the original RCT cohort, which enrolled infants with early FGID symptoms (e.g., colic, regurgitation, constipation), thereby representing an enriched population at higher risk. Additional factors, including early-life antibiotic exposure, diet, and later BMI, may influence long-term functional abdominal pain through effects on gut microbiota, intestinal permeability, inflammation, and gut–brain signaling [12]. Psychosocial stressors and adverse life events can affect hypothalamic–pituitary–adrenal (HPA) axis activity and pain perception, potentially worsening functional symptoms [13]. Likewise, recurrent gastrointestinal or systemic infections during childhood may trigger persistent immune activation or lead to post-infectious functional syndromes [14]. The absence of longitudinal data on these variables limits the ability to disentangle their individual and combined contributions, raising the possibility that cumulative environmental influences over time may partially account for the observed association rather than the early intervention alone. Most probiotic trials in pediatric FGIDs have focused on symptom management after disease onset [9], whereas the present findings are consistent with a potential preventive, disease-modifying approach. However, because no microbiome or immune biomarkers were collected, mechanistic interpretations remain speculative and should be regarded as plausible explanatory models rather than confirmed pathways. The observed association suggests that early-life microbial modulation may extend beyond infancy. Unlike interventions targeting symptom control after disease onset, this approach aligns with a preventive paradigm that aims to modify early risk factors before chronic pain pathways are established [15]. The original trial demonstrated short-term benefits, including reductions in acute symptoms such as colic and regurgitation, which are commonly attributed to transient alterations in gut motility or gas production [16]. The persistence of the association a decade later suggests mechanisms beyond temporary symptom relief, potentially involving developmental processes affecting the gut, immune system, and enteric nervous system. Experimental evidence indicates that *Lactobacillus reuteri* DSM 17938 may modulate host physiology by influencing inflammatory signaling, enhancing mucosal barrier function, and interacting with vagal afferent pathways involved in pain modulation [15]. Early exposure to such signals may contribute to long-term regulation of gut–brain communication and reduced susceptibility to visceral hypersensitivity, a key feature of functional abdominal pain [12,13,14,17]. Early microbiota modulation may also affect barrier integrity, low-grade inflammation, and microbial metabolite production, supporting the hypothesis that early microbial interventions can influence long-term gut–brain axis function [12,13,14,17]. The concept of microbial programming provides a useful framework, proposing that early microbial exposures influence immune tolerance, stress responsiveness, and sensory processing, with effects that persist into later life [18]. Disruptions during critical windows may increase vulnerability to functional disorders, whereas targeted interventions may enhance resilience. These findings align with prior evidence linking early probiotic exposure to neurodevelopmental outcomes, further supporting the role of the microbiota in host system maturation [3]. In this cohort, early feeding type (breast milk versus formula) was not associated with differences in FAP prevalence. However, this subgroup analysis was exploratory and not powered to detect modest differences. The limited sample size within feeding categories reduces statistical power and increases the risk of type II error. Therefore, the absence of a significant association should not be interpreted as evidence of no effect, but rather as an observation requiring confirmation in larger studies. These findings suggest that probiotic supplementation may exert effects partly independent of feeding modality. Although confirmation in adequately powered cohorts is necessary. Several limitations should be acknowledged. FGIDs are influenced by multiple factors—including diet, stress, infections, antibiotic exposure, BMI, psychosocial variables, and other environmental influences—which were not assessed. Additionally, the relatively small size of the breastfed and formula-fed subgroups limited statistical power. Neither the original randomized trial nor the present 10-year follow-up included biological or microbiome measurements, limiting mechanistic interpretation. The absence of longitudinal microbiome data prevents evaluation of whether early probiotic supplementation results in persistent microbial signatures or whether transient changes during critical periods mediate long-term effects. Substantial attrition is an inherent challenge in long-term follow-up studies [19,20]. In the present study, responders had a lower baseline symptom burden than non-responders, suggesting that early symptom severity may have influenced participation and potentially affected prevalence estimates and generalizability. However, follow-up participation was not associated with treatment allocation or sex, indicating that the responder cohort remained broadly representative of the original randomized population. Selective participation based on symptom burden cannot be excluded and may have influenced the observed prevalence of FAP. Although the original intervention was conducted within a randomized controlled trial, the present follow-up is observational, and causal inferences regarding long-term preventive effects cannot be established. Therefore, the findings should be interpreted as evidence of a strong, biologically plausible association rather than proof of causality. Currently, there is insufficient evidence to support routine probiotic use for FGID prevention, as existing studies primarily focus on treatment rather than prevention and exhibit considerable heterogeneity. Nevertheless, these data contribute to the growing literature suggesting that selected probiotic strains, when administered during specific developmental windows, may have disease-modifying potential. If a short course of a targeted probiotic can influence long-term risk of a chronic condition such as FAP, the implications for quality of life and healthcare utilization could be substantial. Finally, the absence of additional adjusted analyses, sensitivity analyses, or multiple testing corrections reflects limitations in data availability rather than a lack of analytical intent. It should be considered when interpreting the robustness of the associations.

## 5. Conclusions

This 10-year follow-up study identifies an association between early-life administration of *Lactobacillus reuteri* DSM 17938 and a lower risk of functional abdominal pain (FAP) in childhood. However, due to the observational nature of the follow-up, the high attrition rate, the lack of adjustment for time-varying confounders over the 10-year period, and the absence of microbiome or biomarker data, causality cannot be established. The findings align with the concept of microbial programming, although this interpretation should be approached with caution. Future research should move beyond epidemiological associations to investigate underlying mechanisms, including the persistence of microbial signatures, fecal biomarkers such as calprotectin and short-chain fatty acids, and systemic inflammatory markers. These results underscore the potential relevance of early-life gut microbiota in shaping long-term gut–brain interactions, while highlighting the need for comprehensive and mechanistically informed studies. Rather than supporting immediate clinical application, the findings suggest that early modulation of the gut microbiota may influence long-term gastrointestinal outcomes. Further long-term trials integrating clinical outcomes with microbiome, immune, and neurodevelopmental measures are needed to clarify causal pathways and identify subgroups most likely to benefit. Given the limited data on diet, stress, BMI, infections, psychosocial factors, and the potential for attrition bias, caution is warranted when generalizing these results. Although speculative, the data support the notion that preventive strategies based on early microbial modulation could complement existing management approaches, potentially reducing the long-term burden of FGIDs and improving childhood health trajectories.

## Figures and Tables

**Table 1:** Baseline demographic and clinical characteristics of participants who completed the 10-year follow-up.

Characteristic	*L. reuteri* (99, 49.5%)	Placebo (101, 50.5%)	*p* Value
Male/female sex, No (%)	Male: 49 (49.5%) Female: 50 (50.5%)	Male: 41 (40.6%) Female: 60 (59.4%)	0.261 (ns)
Breast/Formula feeding, No (%)	Breast: 45 (50%) Formula: 45 (50%)	Breast: 46 (49.5%) Formula: 47 (50.5%)	1.000 (ns)
Diagnosed with FAP, No (%)	13 (13.8%)	81 (81.8%)	<0.001

**Table 2:** Baseline infant characteristics of participants who completed the 10-year follow-up (responders) versus those lost to follow-up (non-responders). Data are presented as n (%) or median [interquartile range]. Categorical variables were compared using chi-square tests; continuous variables were compared using Mann–Whitney U tests.

Baseline Characteristic	Responders (n = 200)	Non-Responders (n = 268)	*p*-Value
Original allocation—*L. reuteri* DSM 17938, n (%)	99 (49.5)	139 (51.9)	0.57
Original allocation—Placebo, n (%)	101 (50.5)	129 (48.1)	
Sex—Male, n (%)	90 (45.0)	128 (47.8)	0.60
Sex—Female, n (%)	110 (55.0)	140 (52.2)	
Infant feeding—Breastfed/partially breastfed, n (%)	91 (45.5)	99 (36.9)	<0.001
Infant feeding—Formula-fed, n (%)	92 (46.0)	102 (38.1)	
Infant feeding—Missing, n (%)	17 (8.5)	67 (25.0)	
Colic episodes (number), median [IQR]	2.0 [1.0–3.0]	3.0 [2.0–4.0]	<0.001
Regurgitation episodes (number), median [IQR]	2.0 [1.0–3.0]	2.0 [1.0–3.0]	0.39
Crying time (minutes/day), median [IQR]	65.0 [50.0–80.0]	70.0 [55.0–85.0]	0.005
Pediatrician visits (number), median [IQR]	2.0 [1.0–3.0]	2.0 [1.0–3.0]	0.46
Emergency department visits (number), median [IQR]	0.0 [0.0–1.0]	0.0 [0.0–1.0]	0.77
Specialist visits (number), median [IQR]	0.0 [0.0–1.0]	0.0 [0.0–1.0]	0.22

**Table 3:** Functional abdominal pain at 10 years by treatment group (complete-case analysis).

Treatment Group	FAP Yes	FAP No	Total
*L. reuteri* DSM 17938	13	81	94
Placebo	81	18	99

**Table 4:** Exploratory multivariable logistic regression for functional abdominal pain at 10 years (complete-case analysis).

Predictor	Adjusted OR	95% CI	*p*-Value
*L. reuteri* DSM 17938 (vs. placebo)	0.04	0.01–0.12	<0.001
Male sex (vs. female)	0.80	0.34–1.87	0.60
Formula feeding (vs. breast/partial)	1.10	0.48–2.54	0.82
Feeding missing (vs. breast/partial)	0.90	0.22–3.72	0.89
Colic episodes (number)	1.06	0.84–1.35	0.62
Regurgitation episodes (number)	1.02	0.80–1.30	0.87
Crying time (minutes/day)	1.00	0.99–1.02	0.40

## Data Availability

The datasets generated and analyzed during the current study are not publicly available due to [motivation, e.g., privacy or institutional restrictions] but are available from the corresponding author on reasonable request.

## Abbreviations

FGIDs	Functional Gastrointestinal Disorders
FAP	Functional Abdominal Pain
*L. reuteri*	**Lactobacillus reuteri*
SCFAs	Short-Chain Fatty Acids
BMI	Body Mass Index

## References

[1] Liss D., Paine P., Baber W., Whitehead W.E. (2014). Functional gastrointestinal disorders in childhood and adolescence: A review of the literature. Am. J. Gastroenterol..

[2] Sperber A.D., Bangdiwala S.I., Drossman D.A., Ghoshal U.C., Simrén M., Tack J., Whitehead W.E., Dumitrascu D.L., Fang X., Fukudo S. (2021). Worldwide prevalence and burden of functional gastrointestinal disorders, results of the Rome Foundation Global Study. Gastroenterology.

[3] Tamburini S., Shen N., Wu H.C., Clemente J.C. (2016). The microbiome in early life: Implications for health outcomes. Nat. Med..

[4] Thompson W.G., Heaton K.W., Smyth G.T., Shine P. (2012). Irritable bowel syndrome in the community: Prevalence, consultation patterns and relationship to lifestyle variables. Gut.

[5] Milani C., Duranti S., Bottacini F., Casey E., Turroni F., Mahony J., Belzer C., Delgado Palacio S., Arboleya Montes S., Mancabelli L. (2017). The First Microbial Colonizers of the Human Gut: Composition, Activities, and Health Implications of the Infant Gut Microbiota. Microbiol. Mol. Biol. Rev..

[6] Hill C., Guarner F., Reid G., Gibson G.R., Merenstein D.J., Pot B., Morelli L., Canani R.B., Flint H.J., Salminen S. (2014). Expert consensus document. The International Scientific Association for Probiotics and Prebiotics consensus statement on the scope and appropriate use of the term probiotic. Nat. Rev. Gastroenterol. Hepatol..

[7] Pärtty A., Kalliomäki M., Salminen S. (2015). Probiotics and the developing brain. Gut Microbes.

[8] Indrio F., Di Mauro A., Riezzo G., Cavallo L., Francavilla R. (2015). Infantile colic, regurgitation, and constipation: An early traumatic insult in the development of functional gastrointestinal disorders in children?. Eur. J. Pediatr..

[9] Dargenio V.N., Cristofori F., Dargenio C., Giordano P., Indrio F., Celano G., Francavilla R. (2022). Use of *Limosilactobacillus reuteri* DSM 17938 in paediatric gastrointestinal disorders: An updated review. Benef. Microbes.

[10] Indrio F., Di Mauro A., Riezzo G., Civardi E., Intini C., Corvaglia L., Ballardini E., Bisceglia M., Cinquetti M., Brazzoduro E. (2014). Prophylactic use of a probiotic in the prevention of colic, regurgitation, and functional constipation: A randomized clinical trial. JAMA Pediatr..

[11] Zeevenhooven J., Koppen I.J., Benninga M.A. (2017). The New Rome IV Criteria for Functional Gastrointestinal Disorders in Infants and Toddlers. Pediatr. Gastroenterol. Hepatol. Nutr..

[12] Bischoff S.C., Barbara G., Buurman W., Ockhuizen T., Schulzke J.-D., Serino M., Tilg H., Watson A., Wells J.M., Pihlsgård M. (2014). Intestinal permeability—A new target for disease prevention and therapy. BMC Gastroenterol..

[13] Mayer E.A., Tillisch K. (2011). The gut–brain axis in abdominal pain syndromes. Annu. Rev. Med..

[14] Belkaid Y., Hand T.W. (2014). Role of the microbiota in immunity and inflammation. Cell.

[15] Vandenplas Y., Benninga M., Broekaert I., Falconer J., Gottrand F., Guarino A., Lifschitz C., Lionetti P., Orel R., Papadopoulou A. (2016). Functional gastro-intestinal disorder algorithms focus on early recognition, parental reassurance and nutritional strategies. Acta Paediatr..

[16] Peng Y., Ma Y., Luo Z., Jiang Y., Xu Z., Yu R. (2023). *Lactobacillus reuteri* in digestive system diseases: Focus on clinical trials and mechanisms. Front. Cell. Infect. Microbiol..

[17] Silva Y.P., Bernardi A., Frozza R.L. (2020). The role of short-chain fatty acids in the interplay between gut microbiota and the brain. Front. Endocrinol..

[18] Mogoş G.F.R., Manciulea Profir M., Enache R.M., Pavelescu L.A., Popescu Roşu O.A., Cretoiu S.M., Marinescu I. (2025). Intestinal Microbiota in Early Life: Latest Findings Regarding the Role of Probiotics as a Treatment Approach for Dysbiosis. Nutrients.

[19] Marmot M., Brunner E. (2005). Cohort Profile: The Whitehall II study. Int. J. Epidemiol..

[20] Dobson A.J., Hockey R., Brown W.J., Byles J.E., Loxton D.J., McLaughlin D., Tooth L.R., Mishra G.D. (2015). Cohort Profile Update: Australian Longitudinal Study on Women’s Health. Int. J. Epidemiol..

